# Frontiers and future perspectives of neuroimmunology

**DOI:** 10.1016/j.fmre.2022.10.002

**Published:** 2022-10-19

**Authors:** Hai Qi, Shumin Duan, Yanying Xu, Hongliang Zhang

**Affiliations:** a School of Medicine, Tsinghua University, Beijing 100084, China; b Faculty of Medicine and Pharmaceutical Sciences, Zhejiang University, Hangzhou 310014, China; c Department of Life Sciences, National Natural Science Foundation of China, Beijing 100085, China

**Keywords:** Neuroimmunology, Neuroimmune interplay, Neuro-endocrine-immune network, Basic research, Key scientific questions

## Abstract

Neuroimmunology is an interdisciplinary branch of biomedical science that emerges from the intersection of studies on the nervous system and the immune system. The complex interplay between the two systems has long been recognized. Research efforts directed at the underlying functional interface and associated pathophysiology, however, have garnered attention only in recent decades. In this narrative review, we highlight significant advances in research on neuroimmune interplay and modulation. A particular focus is on early- and middle-career neuroimmunologists in China and their achievements in frontier areas of "neuroimmune interface", "neuro-endocrine-immune network and modulation", "neuroimmune interactions in diseases", "meningeal lymphatic and glymphatic systems in health and disease", and "tools and methodologies in neuroimmunology research". Key scientific questions and future directions for potential breakthroughs in neuroimmunology research are proposed.

## 1 Introduction

**Fig. 1:**
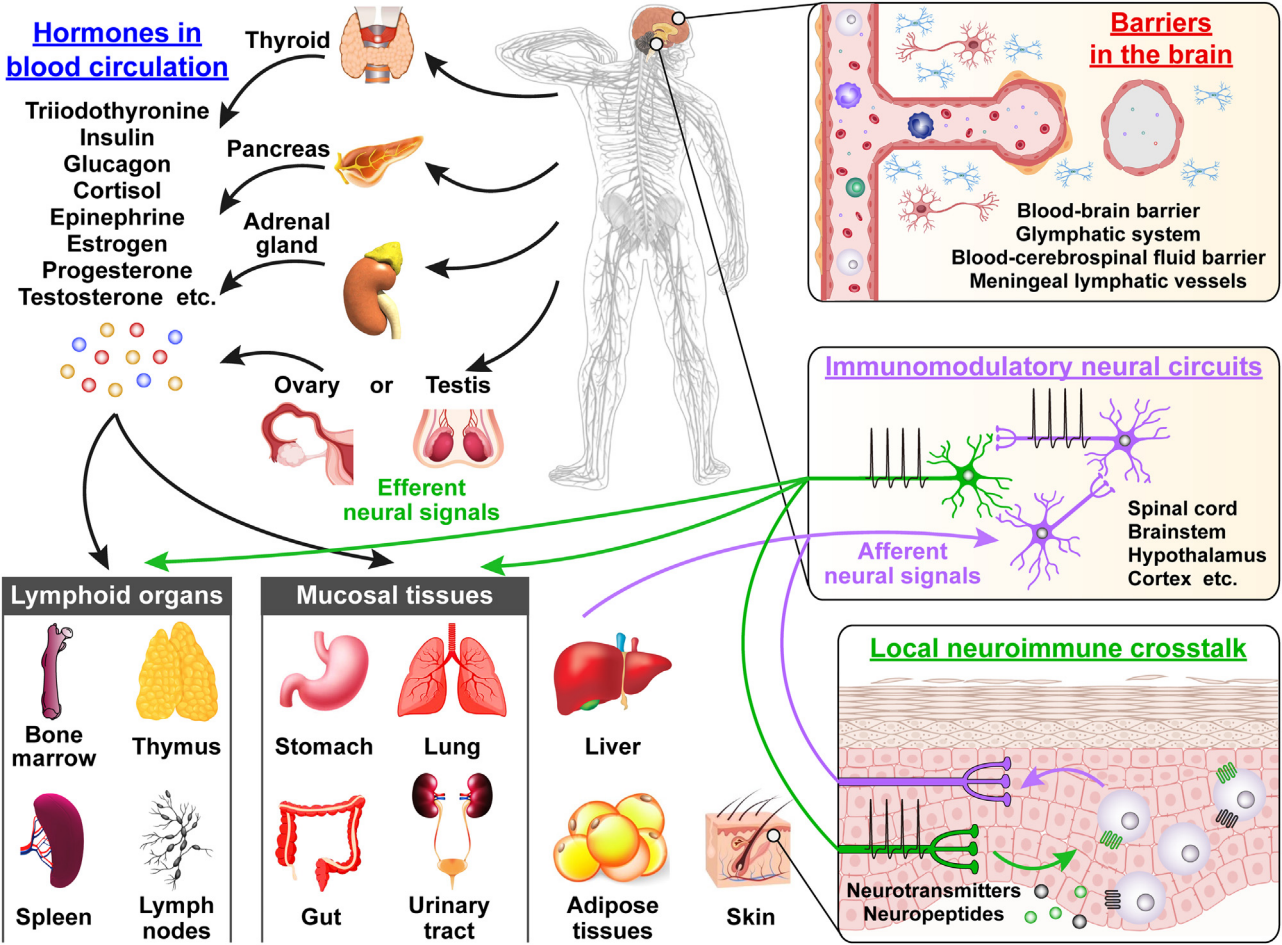
**Functional links between the nervous and the immune systems.** The neuroimmune network in the body consists of conceptually distinct but functionally linked components. First, hormones (*e.g.* triiodothyronine, insulin, glucagon, cortisol, epinephrine, estrogen, progesterone, and testosterone) are released in the blood circulation from endocrine organs (*e.g.* thyroid, pancreas, adrenal glands, ovary, or testis) and exert broad immunomodulatory actions. The neuro-endocrine-immune mechanism may not distinguish among organs or tissues (*e.g.* lymphoid organs, barrier tissues, and metabolic organs). Second, neural innervations in a tissue enable bidirectional neuroimmune crosstalk. Afferent (*i.e.* sensory) signals transduce the status of local immunity to engage different brain regions, and efferent (*i.e.* sympathetic and parasympathetic) signals influence immune responses via neurotransmitters and neuropeptides, establishing tissue-specific neuroimmune circuits. Third, the interface between the brain and the peripheral immune system is enacted by several barrier structures (*e.g.* blood-brain barrier, the glymphatic system, and meningeal lymphatic vessels). Functional interactions between glial cells (*e.g.* microglia and astrocytes) and peripheral immune cells designate immune homeostasis of the brain. (Illustration by courtesy of Professor Jing Yang from Peking University).

The modes of functional crosstalk between the nervous and immune systems could be exemplified in the following three categories (Fig. 1). (1) Structural units named "neuroimmune units" in tissues and organs. The similarities of intercellular communications are readily observed between the nervous and immune systems. For instance, cells in both the nervous and immune systems could communicate through physical connections, forming neuronal or immunological synapses, respectively. A neuronal synapse is a nexus through which two neurons transit information, while an immunological synapse is a dynamic interface between T cells and antigen-presenting cells established by innate and adaptive immunoreceptors, ligands, and adhesion molecules. Neuronal synaptic transmission relies on neurotransmitters or neuromodulators released from presynaptic structures, whereas immunological synapses depend on the membrane-bound molecular recognition between T cells and antigen-presenting cells. During both neuronal and immunological synaptic transmissions, Ca^2+^ influx triggers release of neurotransmitters and activation of immune cells. This shared set of principles have to a great extent facilitated the generation of much-needed interdisciplinary notions such as the “neuroimmune units” to better define the functional neuroimmune interfaces. Even further, the concept of "neuroimmune synapse" has been coined to describe the direct link between neurons and immune cells, though its precise structural and molecular details await in-depth investigation. The evidence is accumulating to show that tissue immunity is tightly modulated by the central and peripheral nervous systems despite the heterogeneous organ compositions or physiological functions. It is highly likely that the “neuroimmune units” could be assembled by neurons and immune cells far beyond direct contact. Further studies are needed to provide a comprehensive understanding of how the two systems may work coherently in various contexts. (2) Homeostatic regulation of immune responses. The immune reactions such as inflammation and antigen-specific immune responses are physiological processes that help restore tissue homeostasis after injuries or infections, but their dysregulation would trigger many diseases. In particular, both direct and indirect regulation of immune response could occur in lymphoid organs via the descending pathways from the central nervous system (CNS). However, unsolved critical questions remain, *e.g.* the identities of neural circuits instructing immune responses, the sensation of immune status by the nervous system, and immune regulation conditioned by innate or learned behaviors, are largely elusive. (3) Regulation of the homeostasis and functions of the nervous system by immune responses. Various neurophysiological activities, such as learning, memory, and social interaction, rely on the proper action of specific neural circuits. Upon injuries, infections, or autoimmunity, the immune homeostasis in the CNS may be compromised, leading to neuroinflammation and neurological disorders.

This review article focuses on the significant research advances of early- and middle-career neuroimmunologists in China. Representative achievements in five aspects of the field, *i.e.*, "neuroimmune interface", "neuro-endocrine-immune network and modulation", "neuroimmune interactions in diseases", "meningeal lymphatic and glymphatic systems in health and disease", and "tools and methodologies in neuroimmunology research", are summarized, which may pave the way for future research in neuroimmunology.

## 2 Neuroimmune interface

Neuroimmune units are formed by nerve fibers and their interacting immune cells, whose actions are mediated by neurotransmitters, neuropeptides, cytokines, and other effector molecules. Neuroimmune units exist in many tissues throughout the body, such as bone marrow, thymus, spleen, lung, skin, gastrointestinal tract, and brain, which function to coordinate a variety of physiological and pathological processes including immune defense, neuroinflammation, and tumors (Fig. 1).

Microglia are a type of resident immune cells in the CNS, which have the most direct neuroimmune interaction with neurons. Microglia play a key role in various processes, such as neural development, CNS homeostasis, and pathogenesis of CNS disorders. During neural development, microglia facilitate the survival of pyramidal neurons in the layer V of the cortex [1]. Clearance of apoptotic cell debris primarily relies on microglia [2]. Microglia remove redundant dendritic spines via signaling molecules such as the complement Clq [3]. Microglia are constantly renewed, maintaining a steady cell number in the brain [4,5]. Moreover, microglia influence the synaptic modification process related to learning and memory by releasing brain-derived neurotrophic factor (BDNF) [6]. Therefore, microglia exert essential roles in sustaining the structural and functional homeostasis of the CNS. In a diseased brain, microglia are activated and move rapidly to injured sites, acting as a double-edged sword: on one hand, activated microglia promote the repair of damaged neural tissues by producing several neurotrophic factors; on the other hand, microglia may exaggerate neural damage by releasing inflammatory factors [7]. As a result, microglial actions in the CNS are complex and pleiotropic.

Meninges are a crucial barrier tissue between the brain and peripheral parts of the body. Discoveries of the meningeal lymphatic and glymphatic systems and meningeal lymphatic vessels have opened up a new dimension in understanding the transport of immune cells and macromolecules into or out of the brain [8]. In the dural sinuses, CD31^+^ endothelial cells secrete interleukin (IL)-7 to promote the differentiation and selection of naïve B cells [9]. In the cerebrospinal fluid of patients suffering from tumor metastasis to the pia mater, peripheral immune cells would infiltrate and significantly alter the immune microenvironment of the brain, thus facilitating the growth of malignant tumors. In particular, macrophages recruited into the cerebrospinal fluid produce several inflammatory cytokines and trigger the expression of lipocalin-2 in tumor cells for their growth [10]. Moreover, meningeal immune response might be related to learning and memory; in the pyemia-related brain disease condition, the meningeal CD4^+^ T cell infiltration has a protective effect on cognition, while blocking such CD4^+^T cells would cause cognitive impairments [11].

Barrier tissues of the body, including the lung, skin, and gastrointestinal tract, host complex neuroimmune interactions. (1) The lung has sensory and cholinergic parasympathetic innervations onto the airways mostly derived from the vagus nerves. Allergic inflammation in the lung stimulates the production of type 2 cytokine IL-5, which triggers sensory fibers to release vasoactive intestinal peptides (VIP). This neuropeptide then activates type 2 innate lymphoid cells (ILC2s) and CD4^+^ T cells thereby aggravating inflammation of the lung [12]. The transient receptor potential vanilloid (TRPV) 1-positive sensory fibers limit the recruitment of neutrophils and γδT cells by secreting calcitonin gene-related peptide (CGRP) that regulates the IL-5 production of ILC2s [13]. Parasympathetic fibers release neuromedin U, which plays important roles in anti-parasitic immunity through enhancing the production of type 2 cytokines IL-5 and IL-13, as well as amphiregulin [14]. Moreover, local sympathetic innervations release norepinephrine, which functions via the β2-adrenergic receptor expressed by ILC2s to suppress the production of IL-5 and IL-13 [14]. This sympathetic signal inhibits the inflammation by alveolar macrophages to mitigate acute lung injuries caused by bacterial infection [15]. (2) The skin is innervated by sensory fibers from the dorsal root ganglia. Those sensory fibers have been shown to interact with immune cells residing in the skin. During bacterial infections such as *Staphylococcus aureus* or *Streptococcus pyogenes*, the pore-forming toxins activate sensory fibers to release CGRP that inhibits the macrophage production of inflammatory cytokines and thus blocks the recruitment and functions of neutrophils [16,17]. This neuropeptide triggers dermal dendritic cells to produce IL-23, which enhances γδT cells to secret IL-17 for anti-fungal immunity [18]. Allergen proteases can stimulate TRPV1^+^ sensory fibers to release substance P (SP) that acts on dendritic cells to promote their migration into draining lymph nodes for initiating type 2 immune responses. SP promotes the degranulation of mast cells, inducing tissue edema and neutrophil recruitment [19]. Meanwhile, this neuropeptide may cause glutamate release from sensory fibers, which mitigates the mast cell degranulation. Moreover, the specific type of sensory fibers can produce the neuropeptide TAFA chemokine-like family member 4 to switch macrophages into the anti-inflammatory status capable of tissue repairing [20]. (3) The gastrointestinal tract is heavily innervated by sensory fibers and the autonomic nervous system, the latter of which consists of enteric neurons and sympathetic and parasympathetic inputs. Sensory fibers from the vagal ganglia and lumbosacral dorsal root ganglia dominate the gastrointestinal tract. TRPV1^+^ and Nav1.8^+^ sensory fibers could directly respond to the infection of *Salmonella typhimurium* and trigger their release of CGRP, which reduces the number of microfold cells and restricts their passage of *Salmonella typhimurium* [21]. Sensory fibers in the gastrointestinal tract produce VIP that modulates ILC2s and type 3 innate lymphoid cells (ILC3s) to boost their production of effector cytokines [22]. Enteric neurons express the granulocyte colony-stimulating factor for the maintenance of muscularis macrophages. Norepinephrine from the sympathetic inputs induces the neuron-protective function of macrophages during bacterial infection [23]. Moreover, the function of ILC2s in the gastrointestinal tract is modulated by neuromedin U [24]. Finally, multiple neural signals converge onto mast cells to tune the production of several inflammatory mediators [25].

In the past years, scientists in China have made remarkable progress related to the structural and functional features of the neuroimmune interface: (1) discoveries of the mechanisms underlying how the brain-autonomic nerves interact with spleen immunity [26,27]; (2) revelation of the insulin signal-mediated GABAergic neuronal suppression of intestinal innate immunity [28]; (3) identification of the degeneration of hepatic sympathetic nerves that influences metabolic liver damage caused by macrophage tumor necrosis factor (TNF)-α [29]; (4) documentation of the neural control of eosinophilic granulocyte extracellular traps involved in allergic asthma [30]; (5) development of microglia replacement strategies, providing a new entry point for treating neurological diseases [5,31,32] and establishing a theoretical model of microglia-mediated memory maintenance [33].

## 3 Neuro-endocrine-immune network and modulation

The neuroimmune interplay in the CNS has unique features and exerts profound effects on neurophysiological functions [34]. Neuronal activities can affect immune cells in the brain, *e.g.* microglia or resident macrophages [35]. The barrier structures of the brain include the choroid plexus, meninges, circumventricular organs, and the blood-brain barrier (BBB), which are all modulated by neural signals. Such neural signals influence the production of immune factors in the choroid plexus and meninges, alter epithelial cells in circumventricular organs, and control the permeability of the BBB [36]. Patients of depression exhibit abnormalities of the BBB, and suppression of the 5-hydroxytryptamine production could reduce its permeability [37]. Sleep deprivation promotes the CXCL13 expression, triggering the recruitment of B cells from the peripheral circulation [38]. Meanwhile, stress conditions affect the lymph transport through the choroid plexus in a glucocorticoid-dependent manner, and blocking the glucocorticoid-receptor signal would help recruit immune cells such as regulatory T cells and reduce behavioral deficits [39]. Therefore, neural signals modulate resident and infiltrating immune cells while such immune cells also participate in the pathophysiology of the CNS in a reciprocal manner.

Neuroimmune mechanisms widely occur in the CNS and peripheral organs of the body. Neuroactive molecules and metabolites derived from the gastrointestinal tract and its microbes directly or indirectly influence the CNS and contribute to neurological disorders. Research on the enteric nervous system has revealed modulatory effects of the gut-brain axis on the immune system. Regulation of neural signals by CGRP in the intestinal mucosa is crucial for maintaining the homeostasis of tissue immunity [40]. Intestinal hypoxia-inducible factor 2a (HIF-2a) regulates the expression levels of *Ldha*, and alters the balance of gut microbiome, which reshapes the immune microenvironment [41]. Moreover, inflammatory responses induce the abnormal accumulation of α-synuclein in the appendix, producing a Parkinson's disease-like phenotype in the gut [42].

The CNS can instruct peripheral immunity via several mechanisms [43]. (1) Endocrine mechanism: consists of the hypothalamic-neurohypophysial system (oxytocin and arginine vasopressin) and the hypothalamic-hypophyseal portal system (adrenocorticotrophic hormone, thyroid-stimulating hormone, follicle-stimulating hormone, luteinizing hormone, and growth hormone). (2) Sympathetic nervous system: includes the systemic and local sympathetic pathways. The systemic pathway is mediated by the adrenal medulla for the secretion of epinephrine and norepinephrine, whereas the local pathway is enacted by sympathetic inputs to different parts of the body, including lymphoid organs. (3) Parasympathetic nervous system: consists of cholinergic innervations reaching certain organs of the body. Notably, parasympathetic inputs are mostly absent in lymphoid organs. (4) Sensory innervations: modulate immune cells by releasing neuropeptides. At the same time, sensory innervations relay the information of tissue injuries or infections to the brain. (5) Meningeal system: delivers brain-derived macromolecules or immune signals to peripheral immune organs. (6) Peripheral immune cells may produce a variety of neuroactive molecules, *e.g.* short-chain fatty acids, purine nucleotides, and serotonin.

Much progress has been made in the understanding of the functional link between the brain and peripheral immune responses. For instance, corticotropin-releasing hormone-positive neurons in the central nucleus of the amygdala (CeA) and the paraventricular nucleus (PVN) could modulate T cell-dependent and antigen-specific antibody immunity via a descending neural pathway through the splenic nerves [27]. This study reveals brain control of adaptive immunity and suggests the possibility to enhance immunocompetency by behavioral intervention.

Microbes have critical roles in sculpturing the neuro-endocrine-immune network. Intestinal microbes can contribute to immune homeostasis through several mechanisms. The secreted protein ClpB produced by commensal intestinal bacteria such as *Escherichia coli* acts as an analog of the α-melanocyte stimulating hormone (α-MSH). ClpB circulates in the blood and activates specific neurons expressing melanocortin receptor 4 to suppress appetite and obesity [44]. Metabolites of intestinal microbes, *e.g.* bile acid and ceramides, are important signaling molecules, and multiple new targets for the intervention of metabolic diseases have been reported, including the intestinal nuclear receptor farnesoid X receptor, G protein-coupled bile acid receptor, and HIF-1/2α [45,46]. Furthermore, intestinal microbes control food intake and glucose metabolism through CART^+^ neurons and neural circuits involving sympathetic inputs in the liver and pancreas [47]. Intestinal microbes also participate in metabolic diseases by influencing the immune system. They produce short-chain fatty acids, which act via the specific receptors GPR43 and GPR109 to modulate regulatory T cells, macrophages, dendritic cells, and other immune cells for maintaining the intestinal immune microenvironment. Tryptophan derivatives produced by intestinal microbes influence the cell fate of the immune system to mitigate insulin resistance and related metabolic disorders [48,49]. Furthermore, abnormalities of lung microbes result in the CNS susceptibility to autoimmune diseases [50]. By converting lung microbes to lipopolysaccharide-rich groups, microglia in the brain are triggered into the type I interferon-expressing state with a reduced response to type II interferon, which helps lower the infiltration of immune cells and relieve the clinical symptoms of autoimmunity. Conversely, suppressing lipopolysaccharide-rich groups in lung microbes worsens the autoimmune disease in the brain [50], revealing an immunological connection between the lung and brain designated by microbes.

Notably, environmental microbes may participate in settling the body temperature. Research has shown that the higher content of environmental microbes, the higher the body temperature would be [51]. Due to the global implementation of vaccines and antibiotics as well as improved medical services, modern societies observe significantly lower infection rates. As a result, the immune system becomes chronically less active. These factors, combined with the availability of air conditioning and heating, may lead to the gradual, long-term lowering of body tempertature [52].

## 4 Neuroimmune interactions in diseases

The nervous and immune systems perform their functions while modulating each other. Immunological disorders lead to autoimmune diseases in the nervous system, such as multiple sclerosis (MS), neuromyelitis optica spectrum disorder (NMOSD), and autoimmune encephalitis. By contrast, abnormalities of the nervous system cause depression, anxiety, and chronic stress, which induces neuro-endocrine defects to compromise the immune system in the body. In addition, neuroimmune interactions may participate in the onset and progression of neuropathic pain, metabolic syndrome, and other human diseases.

### 4.1 Neuroimmune mechanisms in chronic pain

Pain is an unpleasant sensory and emotional experience associated with, or resembling that associated with, actual or potential tissue damage. Chronic pain is defined as a continuous or repeated occurrence for over three months with an incidence rate reaching 32%−40% among adults aged 18−65 years old in China and exhibits a tendency of increasing onset. Of note is that patients suffering from chronic pain may have psychiatric symptoms such as anxiety and depression, although the mechanism underlying this association is unclear.

**Fig. 2:**
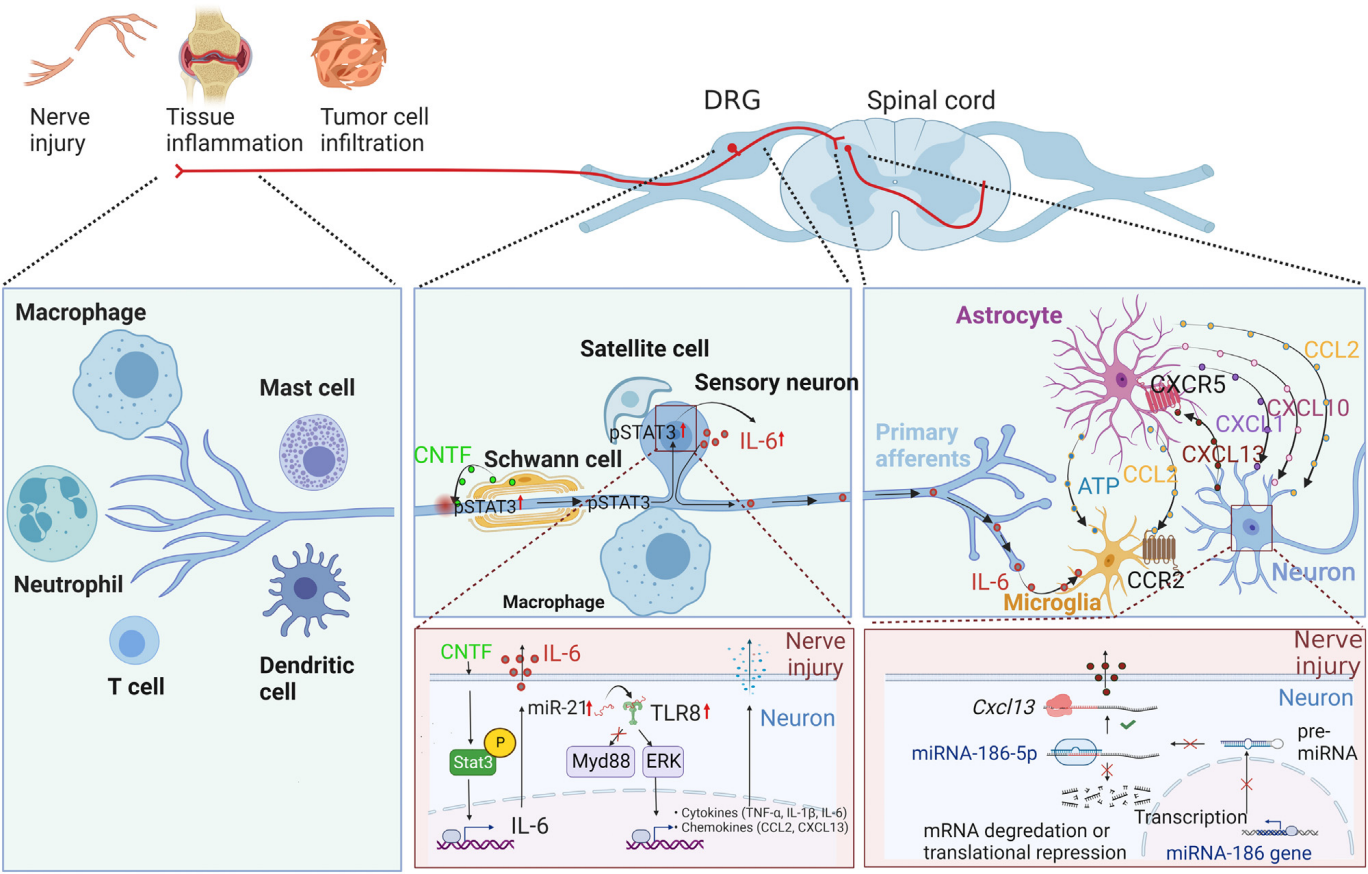
**Neuroimmune mechanisms in chronic pain.** Crosstalk between neurons, microglia, and astrocytes amplifies the pain signal initiated from a periphery site and sustains the duration of pain sensation. Peripheral immune cells (*e.g.* macrophages and T cells) and glia (*e.g.* satellite cells and Schwann cells) affect sensory axons or neuronal cell bodies by releasing inflammatory factors, *e.g.* cytokines and chemokines, to enhance neuronal sensitivity. In the central nervous system, microglia and astrocytes regulate synaptic transmissions via cytokines, chemokines, or neurotrophic factors during chronic pain. Chronic pain alters immune cells and their inflammatory factors in the blood circulation and the cerebrospinal fluid, which may modulate pain sensation. (Illustration by courtesy of Professor Yongjing Gao from Nantong University; the figure was created with BioRender.com) ATP: adenosine triphosphate; CCL: C—C motif chemokine ligand; CCR: C—C chemokine receptor; CNTF: ciliary neurotrophic factor; CXCL: C-X-C motif chemokine ligand; CXCR: C-X-C chemokine receptor; DRG: dorsal root ganglion; IL: interleukin; MyD88: myeloid differentiation factor 88; STAT: signal transducer and activator of transcription; TLR: Toll-like receptor; TNF: tumor necrosis factor.

Although neurons of the peripheral and the central nervous systems have important roles in chronic pain, analgesics targeting neurons often fail to produce satisfactory results. Instead, bidirectional regulation of the immune and nervous systems has a critical involvement (Fig. 2) [53]. Peripheral immune cells (*e.g.* macrophages and T cells) and glia (*e.g.* satellite cells and Schwann cells) affect sensory axons or neuronal cell bodies by releasing inflammatory factors to enhance neuronal sensitivity. In the CNS, microglia and astrocytes regulate synaptic transmissions via cytokines, chemokines, or neurotrophic factors during chronic pain (Fig. 2). Chronic pain alters immune cells and their inflammatory factors in the blood circulation and the cerebrospinal fluid, which may modulate pain sensation. The innate immune pathway of cyclic GMP–AMP synthase (cGAS)-stimulator of interferon genes (STING) contributes to chronic pain. STING-activating molecules could suppress the activity of sensory neurons and achieve pain tolerance [54]. Astrocytes, the most abundant glial cells in the CNS, are actively involved in regulating chronic pain. Nerve injuries increase the production of chemokines (*e.g.* CCL2, CXCL1, and CXCL10) and cytokines (*e.g.* IL-17) in astrocytes of the spinal cord, which promotes the synaptic transmission of excitatory neurons or reduces that of inhibitory neurons. Injured neurons have increased expression of inflammatory factors that influence the functions of astrocytes and microglia.

Studies on immune responses of sensory nerves have shown that nerve injuries increase the expression of ciliary neurotrophic factor (CNTF) in Schwann cells, triggering the IL-6 release from sensory neurons. IL-6 further activates microglia in the spinal cord, which augments neuroinflammation and chronic pain [55]. The expression of Toll-like receptor (TLR) 8 in small-diameter sensory neurons after nerve injury also causes the up-regulation of inflammatory factors via an MyD88-independent way [56]. Interestingly, miR-186 levels are decreased in neurons of the dorsal horn after nerve injury, resulting in increased expression of chemokine CXCL13 that activates astrocytes, leading to the chronic inflammatory response [57]. Such complex interactions between neurons, microglia, and astrocytes amplify the pain signaling from a periphery site and sustain the duration of pain sensation, suggesting new targets for treating chronic pain.

### 4.2 Neuroimmune aspects in endocrine diseases

**Fig. 3:**
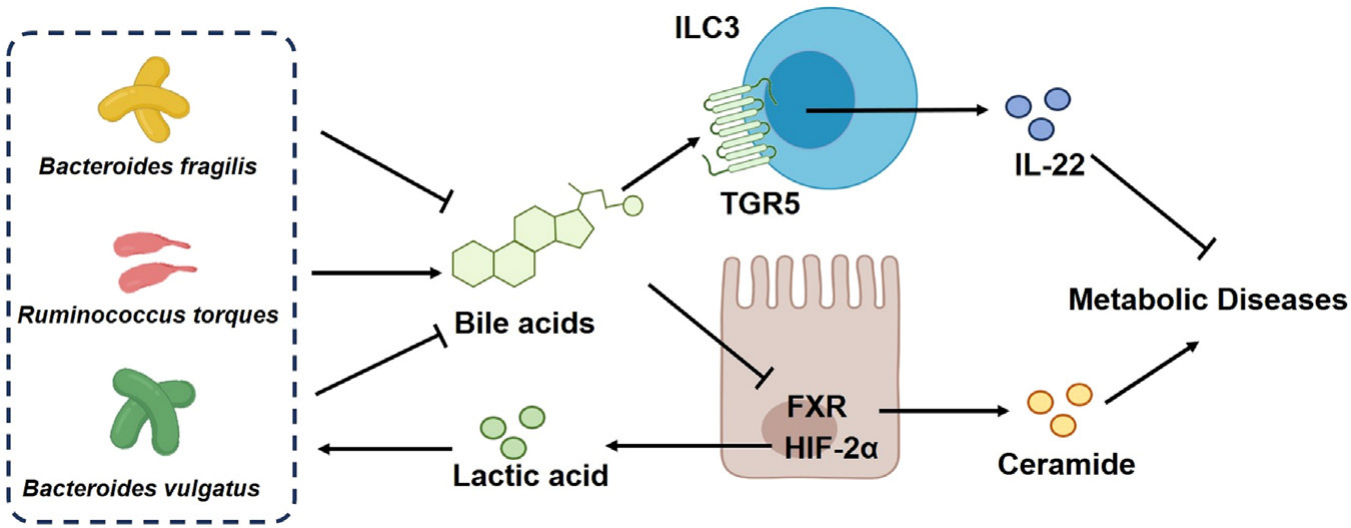
**A theory of "intestinal treatment for metabolic diseases".** Gut microbiota-produced bile acids and ceramide mediates inter-organ crosstalk in the pathogenesis of metabolic diseases. A gut microbiota-bile acid-ILC3-TGR5-IL-22 axis mediates intestinal-ovarian crosstalk to control PCOS. Intestine-derived ceramides, synthesized under the control of FXR and HIF-2α, potentiate metabolic diseases. The bile acid glycoursodeoxycholic acid (GUDCA) was identified as a novel natural FXR antagonist in humans and improved metabolic diseases through the inhibition of intestinal FXR and ceramide signaling. Furthermore, the intestinal HIF-2α induces imbalance of *Bacteroides vulgatus*and *Ruminococcus torques* through the increase of lactate levels, leading to lower DCA and TCA levels, and beiging of white adipose tissue. Intestinal HIF-2α deficiency remodels gut microbiota-bile acid axis to induce thermogenesis and improves metabolic diseases. (Illustration by courtesy of Professor Changtao Jiang from Peking University) FXR: farnesoid X receptor; HIF-2α: hypoxia-inducible factor 2α; IL-22: interleukin-22; ILC3: group 3 innate lymphoid cell; PCOS: polycystic ovary syndrome; TGR5: Takeda G protein-coupled receptor 5.

The neuroimmune network maintains the body's homeostasis while its dysfunction leads to many diseases. ClpB produced by intestinal microbes of obese patients is an α-MSH analog that inhibits appetite and obesity via its action in the CNS [44]. M2-like macrophages in adipose tissues increase fatty-acid metabolism and thermogenesis by producing norepinephrine [58]. In addition, naturally-occurring molecules from plants such as tripterine and withaferin A act on the dorsomedial hypothalamic nucleus to stimulate the sympathetic activity in white adipose tissues, which may increase energy consumption to counter obesity [59]. Furthermore, the nervous and immune systems regulate the constitution and functions of intestinal microbes. Scientists in China have established a series of research platforms and techniques and proposed a theory of "intestinal treatment for metabolic diseases" (Fig. 3), including new modes of intestinal microbes interacting with host immunity and metabolism; metabolites of intestinal microbes as mediators of multiorgan communication, and new therapeutic targets for metabolic diseases [45,46].

Scientists in China have utilized optogenetic approaches to demonstrate how anxiety-related neural circuits trigger osteoporosis. Chronic stress activates inhibitory neurons in the forebrain to modulate neural activities of the hypothalamus and the brainstem, which increases peripheral sympathetic activity. The sympathetic neurotransmitter norepinephrine then acts on osteoblasts to decrease bone density [60]. By developing a calcium-responsive automatic photo-regulation system, scientists achieved the rhythmic control of parathyroid hormone secretion, allowing the intervention of bone loss caused by secondary parathyroid excitation [61]. These results have broadened the application of optogenetics in the bone and endocrine research fields and provided a potential entry point for clinical translation.

### 4.3 Neuroimmune interplay in neurological diseases

CNS was previously considered to be an immune-privileged site. However, peripheral immune cells may infiltrate into the brain upon various pathological insults and participate in neurological diseases. Indeed, accumulating evidence has indicated that aberrant immune responses contribute to most disease conditions of the CNS.

**Fig. 4:**
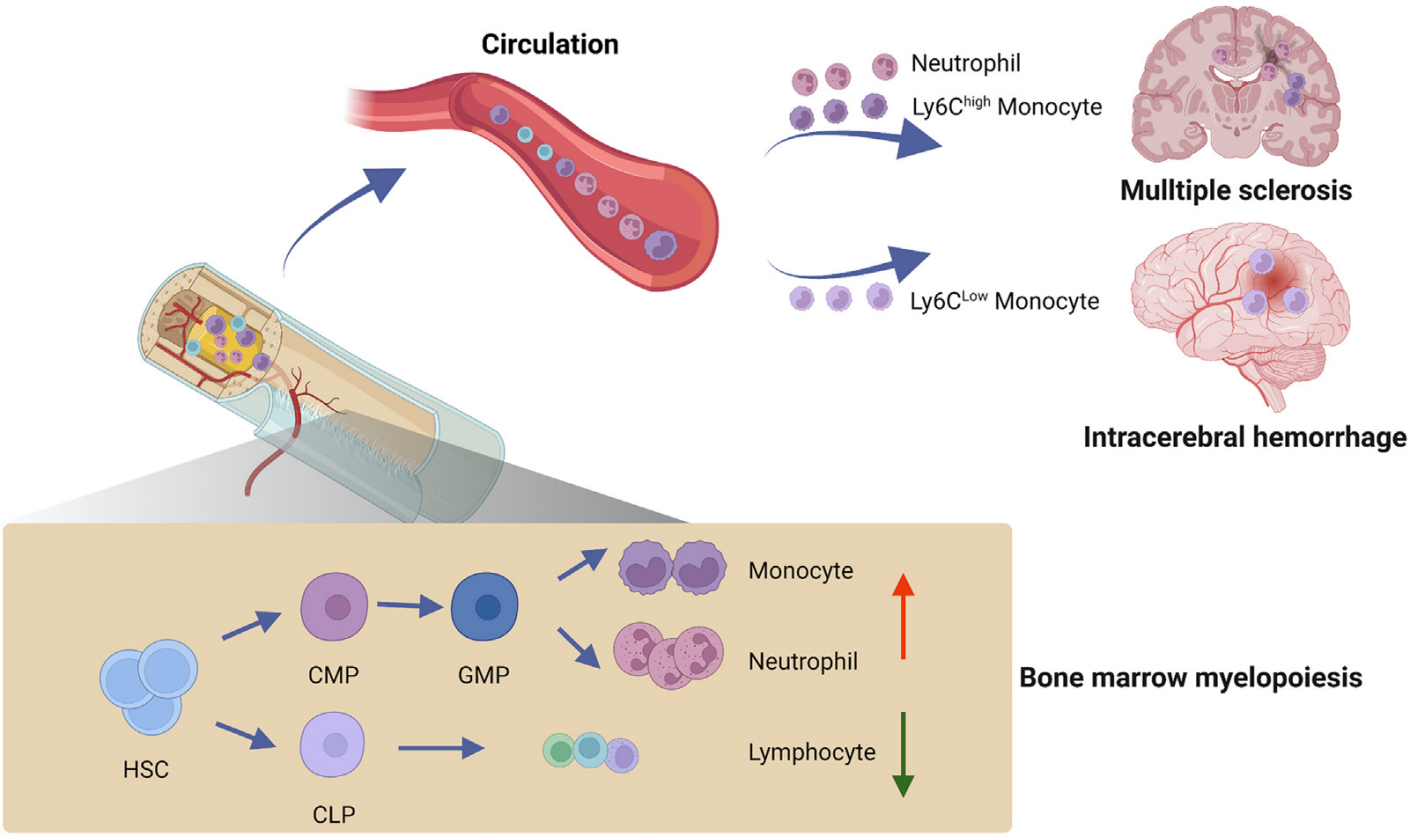
**Bone marrow-brain axis: bone marrow myelopoiesis in brain disorders.** Comprehensive profiling of the bone marrow hematopoietic stem and progenitor cells identified a previously unrecognized role of aberrant myelopoiesis in multiple sclerosis and intracerebral hemorrhage. Under these conditions, bone marrow hematopoietic stem and progenitor cells are skewed toward myeloid lineages, leading to augmented output of meyloied cells that control CNS inflammation and disease progression. (Illustration by courtesy of Professor Qiang Liu from Tianjin Medical University General Hospital) CLP: common lymphoid progenitor cell; CMP: common myeloid progenitor cell; GMP: granulocyte-monocyte progenitor cell; HSC: hematopoietic stem cell.

Autoimmune encephalitis is one of the common autoimmune diseases in the CNS. Its major symptoms include multifocal or diffuse brain damage, leading to mental disorders, epileptic seizures, and cognition deficits. Autoimmune encephalitis is caused by autoantibodies against N-methyl-d-aspartate receptor (NMDAR), α-amino-3‑hydroxy-5-methyl-4-isoxazole-propionic acid receptor (AMPAR), leucine-rich glioma inactivated 1 (LGI1), contactin-associated protein 2 (CASPR2), γ-aminobutyric acid type A receptor (GABAAR), γ-aminobutyric acid type B receptor (GABABR), *etc.* [62]. Scientists in China have launched a series of preclinical and clinical studies aimed at characterizing these autoantibodies, reducing autoreactive B cells or plasma cells, or neutralizing the disease-causing autoantibodies to explore new avenues in the treatment of autoimmune encephalitis and related disease conditions [63,64]. MS is an autoimmune disease afflicting approximately 2.8 million people worldwide. MS occurs when the immune system attacks myelin sheaths and oligodendrocytes, subsequently leading to axonal degeneration in the CNS. Among CNS-infiltrating immune cells, autoreactive T cells initiate CNS demyelinating lesions, release a cascade of cytokines and chemokines that exacerbate local inflammation, and mobilize hematogenous myeloid cells, most notably neutrophils and monocytes. Currently, it remains unclear how myelin-specific immune responses are initiated and how immune cells penetrate the CNS. Nevertheless, myelin repair might achieve a complete recovery since oligodendrocytes precursor cells (OPCs) are capable of differentiation and myelination and are widely distributed in the CNS for a lifelong time. As the primary site of hematopoiesis, the bone marrow produces all types of immune cells that are responsible for the maintenance of immune homeostasis. However, the dynamic activity of hematopoietic differentiation within the bone marrow niche and its potential impact on MS progression remain unknown. Comprehensive profiling of the bone marrow hematopoietic stem and progenitor cells identified a previously unrecognized role of aberrant myelopoiesis in MS progression; myelin-reactive T cells preferentially migrate into the bone marrow niche and skew the bone marrow hematopoietic stem and progenitor cells toward myeloid lineages through activation of CCL5-CCR5 axis, leading to augmented myelopoiesis, the CNS inflammation, and demyelination (Fig. 4) [65]. These findings suggest that targeting the bone marrow niche presents a promising avenue for future design of treatment for MS and related CNS autoimmune disorders.

Cerebral stroke is caused by the interruption of blood circulation in brain regions. There are 3.3 million cases of stroke annually in China, which imposes heavy economic and social burdens [66]. After a stroke, immunological abnormalities such as immune over-activation or immunosuppression may occur. Microglia in the brain sense damage-associated molecular patterns from dying neurons and become activated, producing a large number of inflammatory factors as the key mechanism of neural damage secondary to stroke. Neuroinflammation in the brain may trigger the hypothalamic-pituitary-adrenal axis and the sympathetic nervous system, which causes the overall suppression of periphery immunity and represents a significant cause of post-stroke infections. Stroke may mobilize hematopoietic stem cells in the bone marrow through the β3-adrenergic receptor-mediated sympathetic signal, stimulating myelopoiesis to mitigate neuroinflammation and cerebral edema (Fig. 4) [67]. Potential new strategies to control neuroinflammation so as to combat post-stroke infections have been suggested [68,69]. Moreover, atherosclerotic plaques cause an inflammatory response in surrounding blood vessels, activating neural fibers in plague sites to send signals to the brain. Further, the CNS influences the progression of atherosclerosis via the sympathetic signal, forming a complete arterial cerebral circuit and revealing a novel role of neural control of cardiovascular diseases [70].

Neurodegenerative diseases, *e.g.* Alzheimer's disease, Parkinson's disease, Huntington's disease, amyotrophic lateral sclerosis, and spinal muscular atrophy, occur with the death of neuronal cell bodies and their axonal structures. Immune responses in the CNS can affect the onset and progression of these neurodegenerative diseases. Activation of microglia leads to chronic neuroinflammation that directly contributes to neuronal death. Mitochondrial stress under neurodegenerative insults triggers the abnormal accumulation of mitochondrial DNA (mtDNA), which activates the innate immune pathway of cGAS-STING. Parkin- or Pink1-deficient mice exhibit mtDNA-related neuroinflammation, and the genetic deletion of STING reduces neuronal loss and relieves symptoms [71,72]. Similarly, in mouse models of Huntington's disease or amyotrophic lateral sclerosis, inhibition or genetic deletion of STING delays the disease progression. Importantly, microglia-derived exosomes promote the spread of clustered α-synuclein in Parkinson's disease [73]. Apart from immune responses in the CNS, periphery immunity modulates neurodegenerative diseases. Intestinal microbes are crucial for the gut-brain axis via their production of lipopolysaccharides, trimethylamines, short-chain fatty acids, and tryptophan. In the Pink1-deficient mice, intestinal Gram-negative bacteria trigger the peripheral and central CD8^+^ T*-*cell activation, inducing the death of dopaminergic neurons to exaggerate Parkinson's disease-like symptoms [74]. In addition, chronic intestinal inflammation results in the increased permeability of the gut, aggravating microglial activation in the CNS to sustain a vicious cycle between the gut and the brain [75]. Moreover, metabolites of intestinal microbes participate in the neuroinflammation during Parkinson's disease, and oral administration of short-chain fatty acids worsens motor deficits [76].

### 4.4 Neuroimmune regulation of tumors

Tumors are the leading cause of death for people under the age of 70 in China [77]. Studies have established that the immune system is critically involved in all steps of tumorigenesis. In particular, the immune microenvironment has a decisive role during tumor growth and metastasis; the neurophysiological status of patients significantly influences the disease process. Recent evidence has confirmed that tumor growth and metastasis could be controlled by neural signals [78]. Neural signals act on tumor cells via neurotransmitters. Conversely, tumor-derived paracrine factors instruct neural activities in the microenvironment. For instance, autonomic nerve fibers inside prostate cancers stimulate the proliferation of blood vessels to sustain tumor growth [79]. Tumor-derived factors circulating the blood may affect normal brain functions as well (*e.g.* sleep), whereas the nervous system can release hormones into the blood circulation to promote or inhibit tumors [78]. Tumors could activate catecholaminergic neurons in the medulla of the brainstem, and ablation of those neurons restores the activity of antitumor CD8^+^ T cells to suppress tumor growth [80].

## 5 Meningeal lymphatic and glymphatic systems in health and disease

Due to the unique anatomical features of the brain such as BBB, and the lack of typical lymphatic vessels, communication routes between the CNS and peripheral immunity have long been debated. The meningeal lymphatic and glymphatic systems [81], as well as meningeal lymphatic vessels [82] have recently been discovered and become an intensive research focus. The meningeal lymphatic and glymphatic systems and meningeal lymphatic vessels provide central pathways for the nervous system-derived macromolecules and immune cells to transport between the cerebrospinal fluid of the brain and cervical lymph nodes. The meningeal lymphatic and glymphatic systems are a highly organized transport system that relies on aquaporin 4 on the "end feet" of astrocytes located along blood vessels. The two systems mediate the exchange between the cerebrospinal fluid and the blood circulation, cleaning metabolic wastes of the brain and maintaining the homeostasis of ions and fluid. Dysfunction of the meningeal lymphatic and glymphatic systems causes the accumulation of neurotoxic molecules and is related to physiological or pathological conditions [81].

Meningeal lymphatic vessels are another essential pathway for conducting the cerebrospinal fluid to cervical lymph nodes [82]. They are evolutionarily conserved in different species, including humans and mice. The function of meningeal lymphatic vessels decreases with aging, which leads to the accumulation of disease-associated molecules, such as amyloid beta peptide (Aβ) and Tau proteins, and correlates with the symptoms of Alzheimer's disease. Meningeal lymphatic vessels also carry brain-derived antigens and serve as a route for immune cells such as CD4^+^ T cells and dendritic cells, exerting critical roles in immune homeostasis or pathogenesis. Scientists in China have pursued research on meningeal lymphatic vessels from the early start of the field, generating animal models and technique platforms. Meningeal lymphatic vessels can respond to cerebral ischemia and penetrate into the injured brain region to form new blood vessels via transdifferentiation. This process helps reconnect blood vessels, which enables the rapid restoration of blood supply in the brain and could be crucial after severe ischemic strokes [83,84]. The Notch signal is essential for the transdifferentiation of meningeal lymphatic vessels and could be inhibited by the EphB4/EphrinB2 signal. Therefore, the EphB4/EphrinB2/Notch axis represents a new therapeutic target for regenerating cerebral blood vessels. In addition, meningeal lymphatic vessels exert antitumor functions in the brain. They facilitate the presentation of tumor-specific antigens to lymph nodes and thus activate antitumor adaptive immunity [8]. Vascular endothelial growth factor (VEGF)-C promotes the development of lymphatic vessels and exhibits beneficial effects for treating brain tumors [8,85]. For instance, the VEGF-C overexpression induces the expansion of meningeal lymphatic vessels and enhances the radiotherapeutic effect in mouse models of brain tumors [86].

## 6 Tools and methodologies in neuroimmunology research

Neuroimmunology research relies on the continuous development and implementation of new technologies. To comprehensively assess the structural basis of neuroimmune network in the body, scientists in China have exploited advanced 3D imaging techniques to visualize neural innervations in organs of different species, including mice, non-human primates, and humans. For instance, by combining the whole-tissue immunolabeling, optical clearing, and lightsheet 3D imaging, the distribution of autonomic innervations in lymphoid organs such as the bone marrow, thymus, spleen, and lymph nodes are documented [87,88], providing the foundation for in-depth investigations of neuroimmune regulation in these immune organs. In addition, 3D imaging studies reveal the detailed features of neural innervations in barrier tissues such as the lung and the gastrointestinal tract of different species [15,89]. Moreover, such advanced imaging power elucidates the structural change to meningeal lymphatic vessels during viral meningitis [90], suggesting a new aspect of the neuroimmune mechanism in meninges.

**Fig. 5:**
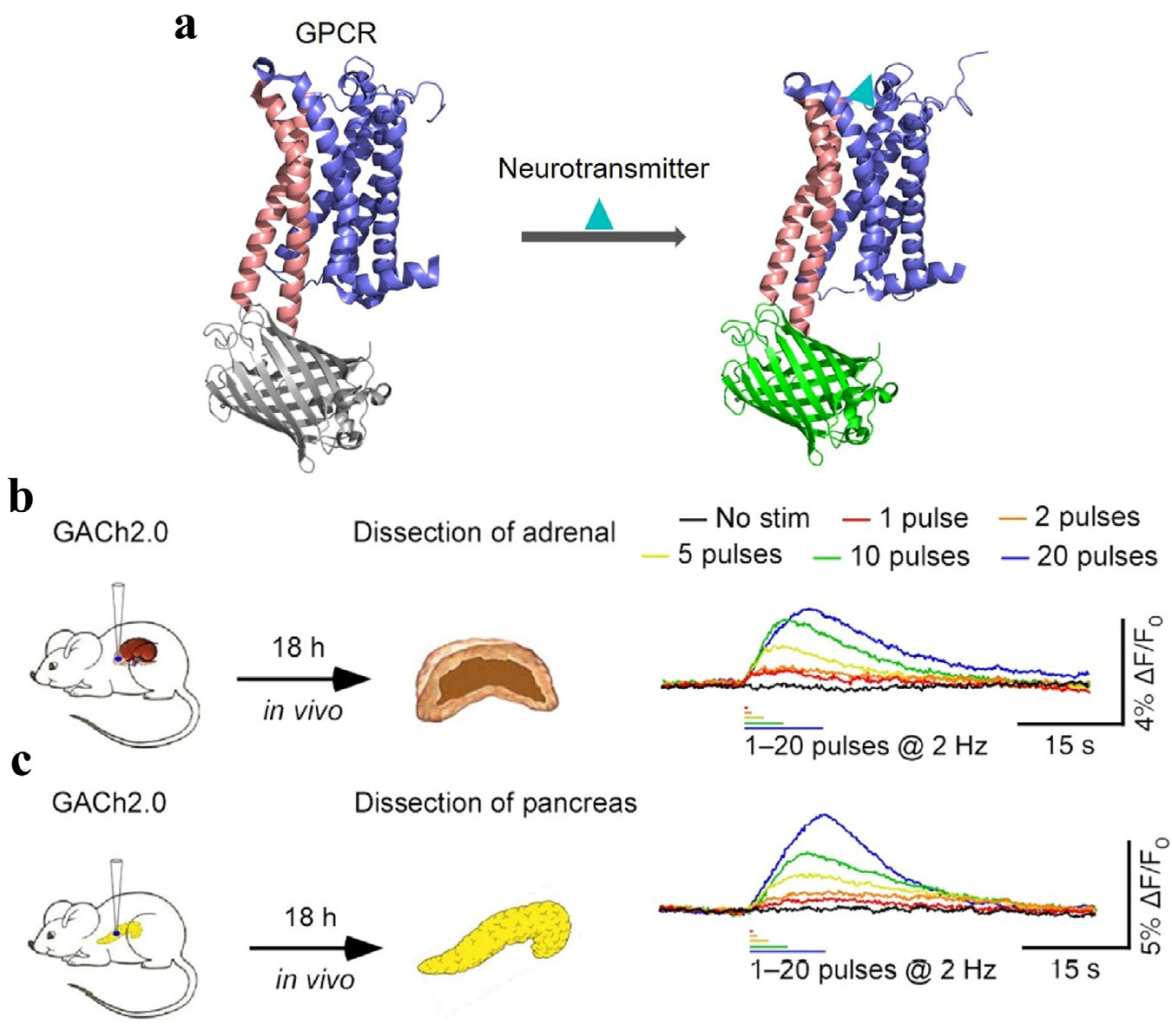
**Principle of GRAB strategy and applications of GRAB**_**ACh**_**sensors in the periphery organs.** GPCRs were linked with the circularly permuted enhanced green fluorescent protein, named GRAB sensors, which help detect different types of neural signals *in vivo*. By using the acetylcholine-specific GRAB sensors, cholinergic signals in the mouse pancreas and adrenal glands are recorded. (Illustration by courtesy of Professor Yulong Li from Peking University) GPCR: G protein-coupled receptor; GRAB: GPCR activation-based.

Recent studies have engineered G protein-coupled receptors (GPCRs) linked with the circularly permuted enhanced green fluorescent protein (cpEGFP), named GPCR activation-based (GRAB) sensors, which help detect different types of neural signals *in vivo*. By utilizing the acetylcholine-specific GRAB sensors, cholinergic signals in the mouse pancreas and adrenal glands are recorded (Fig. 5) [91]. Similarly, dopaminergic [92] or catecholaminegic [93] signals in the murine brain are viewed under physiological or pathological conditions. Moreover, GRAB sensors for adenosine [94] or adenosine triphosphate [95] enable the visualization of immune-related neural signals in the mouse brain. These imaging tools facilitate the detailed characterization of neuroimmune actions in the body.

**Fig. 6:**
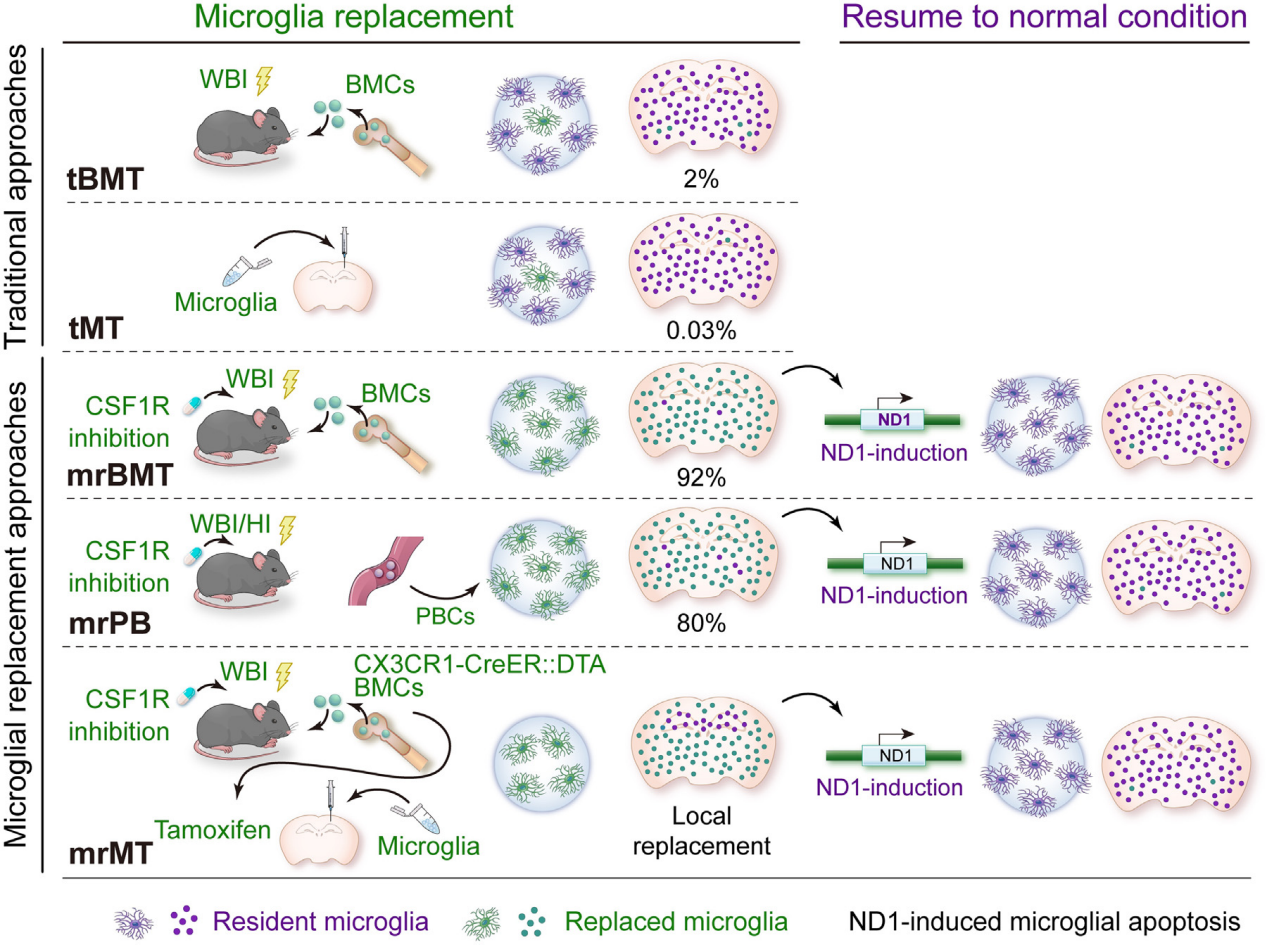
**Efficient strategies for microglia replacement.** Efficient and controllable methods of microglia replacement. High efficiency of allogenic microglial replacement is achieved, providing new therapeutic strategies for neurodegenerative diseases. Based on these new methods, dysfunctional microglia with mutant genes are able to be replaced by normal or gene-corrected cells in the mouse model of neurological diseases. (Illustration by courtesy of Professor Bo Peng from Fudan University) BMCs: bone marrow cells; CSF1R: colony stimulating factor 1 receptor; HI: head irradiation; mrBMT: microglia replacement by bone marrow transplantation; mrMT: microglia replacement by microglia transplantation; mrPB: microglia replacement by peripheral blood; ND1: NeuroD1; PBCs: peripheral blood cells; tBMT: traditional bone marrow transplantation; tMT: traditional microglial transplantation; WBI: whole-body irradiation.

Scientists in China have made advances in microglial replacement for cell therapy-based purposes, which might be clinically feasible [5]. Studies have dissected mechanisms by which the proliferation and migration of microglia are regulated by specific niche and territory-repulsion effect [32]. High efficiency of allogenic microglial relacement is hence achieved based on the understanding of microglial proliferation and migration (Fig. 6) [31,[96], [97], [98]], providing potential therapeutic strategies for neurodegenerative diseases. Furthermore, NeuroD1, which induces glial apoptosis rather than glia-to-neuron conversion, can be utilized as a "molecular brake" for the switch-off control enhancing the safety of microglial replacement [99]. Based on these new approaches, we are able to treat neurological disorders by replacing dysfunctional microglia with gene-normal or gene-corrected allogenic cells in the murine models [100].

**Fig. 7:**
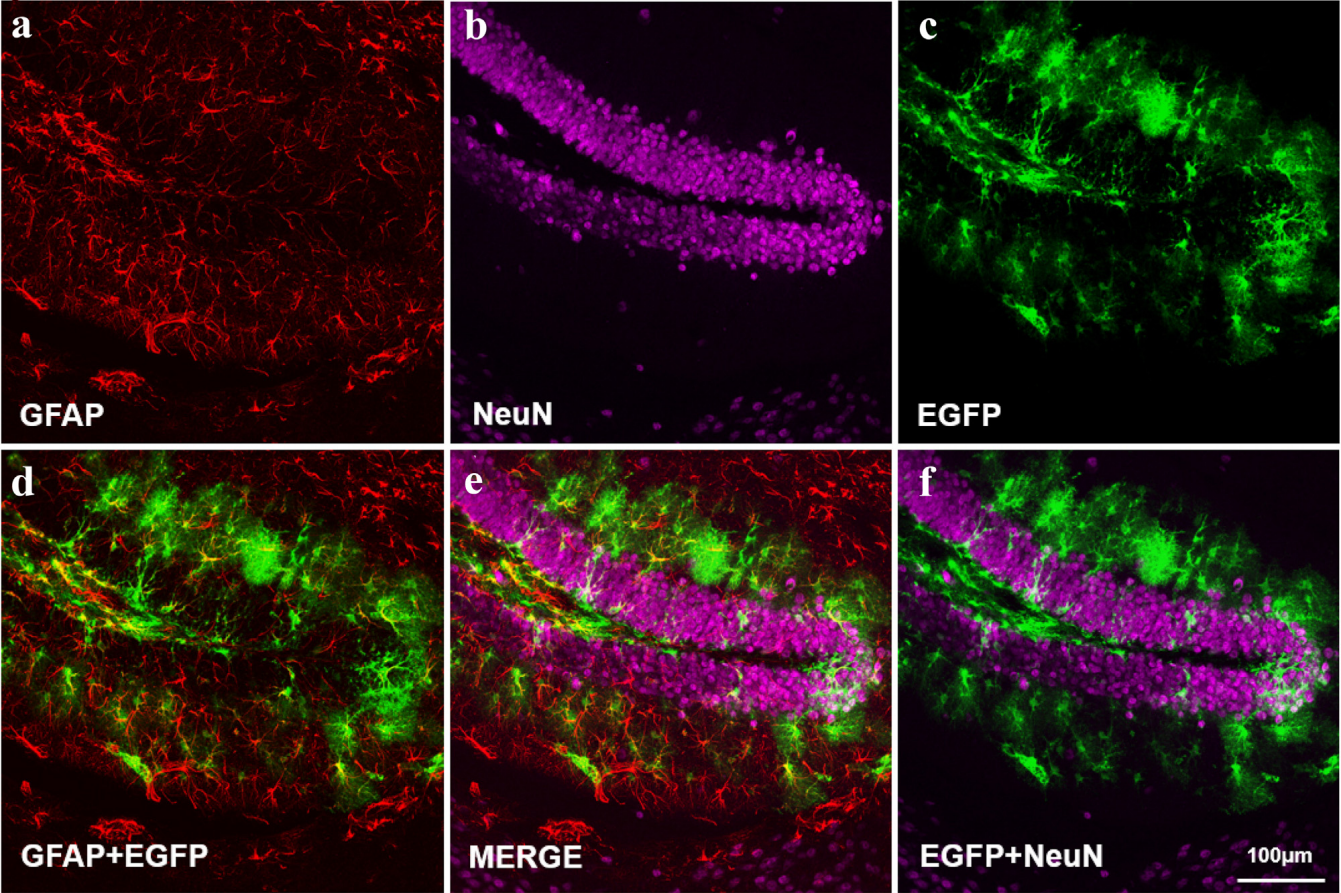
**Efficient transduction of hippocampal astrocytes by the engineered EGFP-expressing adeno-associated virus.** By integrating the approaches of virology, molecular cell biology, neurobiology, bioinformatics, and artificial intelligence, viral vectors are constructed for enhanced labeling efficiency and specificity. Capsid proteins of adeno-associated viruses are systematically mutated and screened to achieve the specific transduction of astrocytes in the brain. These astrocyte-specific viral vectors enable the expression of EGFP and magnetic resonance imaging-compatible aquaporins, which is critical for a direct comparison of mesoscopic and macroscopic hierarchical networks related to immune homeostasis in the brain. (Illustration by courtesy of Professor Fuqiang Xu from Shenzhen Institute of Advanced Technology, Chinese Academy of Sciences) EGFP: enhanced green fluorescent protein; GFAP: glial fibrillary acidic protein; NeuN: neuronal nuclei antigen.

Research efforts have pursued new generations of viral vectors for investigating immune homeostasis in the CNS *in vivo*. By integrating the approaches of virology, molecular cell biology, neurobiology, bioinformatics, and artificial intelligence, over ten types of viral vectors are constructed for enhanced labeling efficiency and specificity but reduced cytotoxicity in neurons [101,102]. Capsid proteins of adeno-associated viruses are systematically mutated and screened to achieve the specific transduction of microglia and astrocytes in the brain [103]. These astrocyte-specific viral vectors enable the expression of EGFP and magnetic resonance imaging (MRI)-compatible aquaporins, which is critical for a direct comparison of mesoscopic and macroscopic hierarchical networks related to immune homeostasis in the brain (Fig. 7**)** [104].

Scientists in China have also explored the metabolic aspect of neuroimmune interplay and establishe technical support for such studies. By developing single-cell mass-spectrum platforms, metabolomics of single neurons could be analyzed accurately, especially for low-concentration and short-lived metabolic intermediates. For example, studies have uncovered a new synthesis pathway of glutamate in neurons, which may participate in learning and cognition functions [105,106]. Moreover, metabolomics of single lysosomes in neurons are examined, revealing the heterogeneity of lysosome metabolism in aging [107].

## 7 Future perspectives

As discussed above, the research community of China has witnessed exciting advances in neuroimmunology and cultivated many excellent early- and middle-career scientists in the field. However, it must be realized that developed countries such as the United States, Germany, and Sweden, still dominate the global research in the neuroimmunology field. Several hurdles are notable for bridging such a gap, including limited funding for basic research, lack of training and cultivation of young researchers, and insufficient attention to technology development. Neuroimmunology research in China should focus on the following key scientific questions for potential breakthroughs in the next 5–10 years:

**(1) Concepts and structure of the neuroimmune network:** Neuroimmune regulation can be achieved through systemic endocrine mechanisms or local neural actions. However, its concepts remain incompletely defined and require further clarifications. (i) Systemic neuro-endocrine-immune regulation: endocrine organs, *e.g.* adrenal glands, thyroid glands, pancreas, and gonads, would collaborate in regulating the body's immunity. Existing studies primarily focus on the immunomodulatory effects of adrenal glands but mostly neglect the potential roles of other endocrine organs. For instance, though evidence has shown that sex hormones significantly affect the progression of many diseases, the neuro-endocrine-immune mechanism underlying such actions of sex hormones is less characterized. Importantly, how endocrine mechanisms would work together with local neural signals to designate the overall outcome of tissue immunity is unclear. (ii) Synergistic effects of neuroimmune crosstalk: Sensory, sympathetic, and parasympathetic innervations in organs can exert diverse immunomodulatory functions. However, how different types of immune cells respond to such neural signals is highly complex. Comprehensive documentation of the collective responses of various immune cells to specific neural signals is crucial for understanding the entire network. (iii) Neuroimmune interplay in the barrier tissues: the skin, the respiratory tract, and the gastrointestinal tract are in direct contact with the external environment. As a result, extrinsic environmental cues collaborate with intrinsic neural signals and immune cells to determine the immunity of these barrier tissues. Therefore, an additional dimension containing commensal microbiota, pathogens, and allergens must be considered in the neuroimmune regulation of barrier tissues. (iv) Neuroimmune mechanisms in the CNS: barrier structures of the CNS are crucial for maintaining immune homeostasis, and their abnormalities contribute to a variety of neurological disorders. However, neuroimmune interactions in the CNS await in-depth investigations. For example, how BBB, the meningeal lymphatic and the glymphatic systems, as well as meningeal lymphatic vessels exert distinct roles in communicating with periphery immunity is not fully understood. How microglia and other resident immune cells in the brain interact with neurons and the barrier structures calls for comprehensive examinations as well.

**(2) Functional links between the neuroimmune network and other systems:** The nervous and immune systems closely interact with other systems in the body, including metabolism, digestion, respiration, circulation, and reproduction. Therefore, crosstalks between the neuroimmune network with other systems fundamentally determine the homeostasis and disease of the body. (i) Neuroimmune control of metabolism: dense neural innervations are present and exert essential roles in metabolic organs, *e.g.* liver, pancreas, and adipose tissues. The systemic characterization of neuroimmune interactions under physiological or disease conditions in these organs is an indispensable part of understanding their relevance in metabolism. In addition, neural signals can influence the digestion and absorption of nutrients such as ions, glucose, and lipids. Neuroimmune actions in the gastrointestinal tract serve as an important metabolic regulator. (ii) Neuroimmune regulation of the circulatory system: the nervous system has critical control of the circulatory system, including heart rate, blood volume, and blood pressure. Neuroimmune mechanims in the circulatory system could extend beyond such classic actions and participate in strokes, myocardial infarction, atherosclerosis, and vesicular inflammation. Exploration of functional links between the neuroimmune network and the circulatory system is of significance for future research.

**(3) Plasticity of the neuroimmune interplay in diseases:** Although studies have examined neuroimmune crosstalk in steady states of the body, structural or functional changes under pathological conditions represent new avenues in understanding the complexity of neuroimmune interplay. (i) Pathological alterations of neural structures and their immunomodulatory effects: the central or peripheral nervous systems suffer damage under various pathological insults such as neural injuries, neurodegeneration, and autoimmune diseases. Such pathological changes may influence the existing neuroimmune network. However, a comprehensive examination of the cellular and molecular mechanism underlying the disease-induced plasticity of neuroimmune interactions warrants future attention. (ii) Neuroimmune regulation under psychological or psychiatric conditions: psychological or psychiatric conditions affect the function and structure of specific neural circuits and profoundly modulate the immune system. However, the mechanisms underlying those observations need to be thoroughly clarified. For instance, the "placebo effect" commonly observed in clinical trials may act through neural circuits related to neuro-endocrine or neuroimmune pathways. (iii) Neuroimmune interplay in tumors: as abnormal tissues, tumors have neuroimmune interactions inside that drastically differ from those in normal organs. Clinical evidence has shown that infiltration of neural fibers into tumors is highly correlated with malignancy. However, how neural signals control tumor growth and metastasis has yet to be fully explored. Research efforts in this direction are particularly relevant for developing effective antitumor therapies.

**(4) Clinical translation of neuroimmunology research:** Neuroimmunology is an interdisciplinary branch of biomedical science, and its research is built upon many clinical observations and evidence. Therefore, the translation of basic research into real-world scenarios should be emphasized. (i) Conservation of neuroimmune mechanisms across species: studies in the field have primarily utilized animal models such as mice and rats. However, it is essential to verify such rodent-derived neuroimmune mechanisms in non-human primates and humans, which is indispensable for any translational effort. (ii) Relevance of animal disease models for clinical translation: A variety of animal disease models have advanced the knowledge of neuroimmune interplay in different organs. However, the intrinsic limitations of such animal models should not be ignored. For instance, studies have shown that microglia in the mouse brain exhibit significant morphological and functional differences compared to human microglia. Similarly, meningeal lymphatic vessels in rodents and humans have prominent anatomical divergences. The relevance of an animal disease model to its corresponding human disease should be validated. (iii) Innovative translational approaches of neuroimmunology: it has now been broadly accepted that the neuroimmune network has a critical role in preventing, treating, and curing human diseases. Clinical translation of neuroimmunology research needs to combine bioengineering, physics, chemistry, and computer science and exploit different methods such as electrical stimulation, magnetic stimulation, and nanomedicine. Moreover, additional insights may be obtained from traditional Chinese medicine (TCM), such as acupuncture and meditation. Taken together, future efforts of neuroimmunology research must tackle key scientific questions with innovative approaches, which would have great implications for human health.

## Declarations

Hongliang Zhang and Yanying Xu are affiliated with the National Natural Science Foundation of China. The views expressed are their own and do not necessarily represent the views of the National Natural Science Foundation of China or the Chinese government.

## Declaration of competing interest

The authors declare that they have no conflicts of interest in this work.
